# COVID-19 preventive measures in Rohingya refugee camps: An assessment of knowledge, attitude and practice

**DOI:** 10.1371/journal.pone.0282558

**Published:** 2024-01-24

**Authors:** Charls Erik Halder, Md Abeed Hasan, Yussuf Mohamed Mohamud, Marsela Nyawara, James Charles Okello, Md Nahid Mizan, Md Abu Sayum, Ahmed Hossain, Andrew Willam, Hamim Tassdik

**Affiliations:** 1 Migration Health Division, International Organization for Migration (IOM), Cox’s Bazar, Bangladesh; 2 College of Health Sciences, University of Sharjah, Sharjah, United Arab Emirates; 3 Global Health Institute, North South University, Dhaka, Bangladesh; University for Development Studies, GHANA

## Abstract

**Background:**

Although many studies were conducted on COVID-19 knowledge, attitude, and practice (KAP) among the general population in many countries, very little is known about refugees, particularly Rohingya refugees in Cox’s Bazar. A vast array of risk communication and community engagement (RCCE) interventions were implemented in Cox’s Bazar with the intent of reducing disease transmission by empowering the community to adopt public health measures.

**Objectives:**

The study aimed to assess the level of knowledge, attitude and practice (KAP) of COVID-19 preventive measures among the Rohingya refugees in Cox’s Bazar, and to identify their socio-demographic determinants.

**Materials and methods:**

A cross-sectional study was conducted with 500 Rohingya individuals. Participants in the study were Rohingya refugees residing in five randomly selected camps where International Organization for Migration (IOM) Health was operating. Using a structured questionnaire, skilled community health workers surveyed the Rohingya population. In addition to the survey on knowledge, attitude, and practice, the study gathered information on the perspectives and relevance of sociodemographic factors that influence KAP.

**Results:**

The study findings indicate that the mean scores for knowledge, attitude, and practice were 9.93, 7.55, and 2.71 respectively. Association was found between knowledge and practice level and age group–the elderly age group (>/ = 61 years) had less level of knowledge (AOR 0.42, P value = 0.058) and the late mid-age group (46–60 years) had better practice level (AOR 2.67, P value <0.001).

**Conclusions:**

Our study found that the Rohingya refugee community in Cox’s Bazar has improved knowledge and attitude toward COVID-19 preventive measures. However, the practice level of these measures remains low compared to the knowledge and positive attitude. The reason behind the poor practice of preventive measures needs to be identified and addressed engaging the community in similar future outbreaks.

## Introduction

The district Cox’s Bazar in Bangladesh is hosting approximately 883,600 Rohingya refugees residing in 34 overcrowded refugee camps following their mass displacement from Myanmar in 2017 [1]. It was preceded in kind by decades of influxes spurned by systematic discrimination and targeted violence in Myanmar [2]. The refugees are living in overcrowded bamboo-made settlements at hilly slopes and basins with limited access to livelihood and basic entitlements and are highly vulnerable to natural and man-made disasters and disease outbreaks [3]. Crowded living conditions, lack of good WASH (Water, Sanitation and Hygiene) facilities and practices, and heavy monsoon in the refugee camps and adjacent host communities increase their susceptibility to infectious diseases and often result in disease outbreaks [4]. Since 2017, several outbreaks or upsurge of infectious diseases, like, diphtheria, measles, AWD/cholera (Acute Watery Diarrhoea) and dengue were reported in the Rohingya camps [4]

Superimposed on the existing vulnerability to disease outbreaks, COVID-19 appeared as a new threat to this population. COVID-19 is a highly contagious emerging disease caused by Severe Acute Respiratory Syndrome Coronavirus 2 (SARS-CoV-2). The disease was first learnt in December 2019 following a report of a cluster of viral pneumonia cases in Wuhan [5]. Since then, the disease continued to spread around the world, with more than 500 million confirmed cases and six million deaths reported across around 200 countries [6]. On March 11, 2020. COVID-19 outbreak was declared by World Health Organization (WHO) as a global pandemic [7]. The first COVID-19-positive case confirmed in Bangladesh was on March 08, 2020, and the first case from the Rohingya refugee camps was reported on May 2020 [8, 9]. As of 24 April 2022, 5,922 COVID-19 confirmed cases (out of 99,049 tests) and 42 deaths were reported from the refugee camps [10]. Public health guidance for prevention and control of COVID-19 included maintaining distancing of one meter from others, wearing a mask, cleaning hands, covering cough and sneezes, getting vaccinated, staying home when sick and seeking medical care when required [11, 12]. Effective implementation of those measures relies largely on what people know about these, how they think or believe about them and how they do or experience these, which in summary is interpreted as knowledge, attitude, and practice (KAP) [13]. Except for a few studies conducted in Pakistan [14] and Jordan [15] respectively among Afan and Syrian refugees, literature on knowledge, attitude and practice regarding COVID-19 preventive measures in the refugee setting is notably sparse. However, a large number of studies were carried out in the field of knowledge, attitude and practices among the general population in a wide range of countries, including, China [16] South Korea [17], Iran [18], Bangladesh [19, 20], Jordan [21], Saudi Arabia [22], Kenya [23] and India [24–27]. While the knowledge, attitude and practice level varied from study to study and country to country, most of the studies found that the level of KAP differs among demographic groups (e.g., age groups, male/females, employment status and income). However, very little is known about the knowledge, attitude, and practice of refugees in humanitarian setting, particularly among Rohingya refugees in Cox’s Bazar.

Before the vaccine was widely available to the population, public health and social measures remained as the most important tool for interrupting disease transmission [28]. Hence, risk communication and community engagement (RCCE) were one of the major pillars of the COVID-19 response strategy [29]. Humanitarian partners, including International Organization for Migration (IOM), in Cox’s Bazar implemented a vast array of RCCE interventions with the intent of reducing disease transmission by empowering the community to adopt public health measures. The interventions included household visits and community meetings by community health workers, dissemination of audio-visual clips, publication of printed materials, social advocacy through social leaders and community groups, go and see visits to the service sites. Most messages and contents of the communication were based on materials developed by Communication with Community (CwC) working group. A KAP survey on COVID-19 carried out among the Rohingya refugees at the very beginning of the outbreak found that the KAP of the respondents were not satisfactory [29]. However, there was no evidence in place regarding the status of the knowledge, attitude and practice among the Refugees after the implementation of the extensive RCCE interventions. Different forums and reports have highlighted the issue of noncompliance among the population with COVID-19 measures; however, there is no evidence as to what extent the public health measures are not accepted or practiced by the community. The findings of the study could support the development of a robust strategy for risk communication and community engagement for the ongoing pandemic as well as future outbreaks of infectious diseases.

## Materials and methods

### Study design and participants

The study was conducted in Cox’s Bazar refugee camps, where the International Organization for Migration (IOM) had implemented community health programs. The population of the Rohingya community in Cox’s Bazar is around 883,600, with a male-to-female ratio of 45:55 [1]. The study was conducted from June to December 2021, coinciding with the period of the delta wave of the COVID-19 pandemic. Notably, a large portion of the population resides in makeshift shelters. It was a cross-sectional study. Rohingya refugees in the selected camps who were 18 years or above were the study participant. The inclusion criteria for this study were that participants must reside inside the camps, be 18 years or older, consist of both male and female participants who give their consent, and must be able to understand the questioner, while the exclusion criteria were that participants cannot belong to the same family. Excluding participants from the same family in this study was a measure to uphold the statistical independence of data and mitigate potential bias. A survey was employed among 500 Rohingya individuals selected through systemic random sampling. 5 camps of IOM health operation were selected randomly. In each camp, 5 sub-blocks were randomly selected and at each sub-block, 20 households were selected through systemic random sampling. At each camp 5 CHWs surveyed 100 beneficiaries of different ages from the selected households; from each household, a beneficiary was selected using an age-sex table prepared based on camp demographic data.

### Sample size calculation

This was a cross-sectional study, given the considerable population size surpassing 20,000, we are employing the "Rule of Thumb for Sample Size" to compute the required sample size. This approach provides a simplified estimation method tailored for large populations. The following formula used for sample size collection: $n=\frac{z^{2}pq}{d^{2}}$

The equation represents a sample size calculation for our study [30]. The variable "q" is calculated as 1-p, where "p" is the population proportion, which is assumed to be 50%. The variable "z" represents the confidence level of interest, which is set at 1.96 for a 95% confidence level. The variable "d" represents the degree of accuracy required, which is set at 0.05 level for the expected sample size. Using these values, the sample size "n" is calculated using the equation (z^2*p* q) / d^2, resulting in a sample size of approximately 384. However, to proactively account for the potential of a 25% non-response rate, this figure is judiciously rounded up to 500, signifying a strategic buffer that fortifies the study’s validity. It represents a methodical approach aimed at ensuring the study’s outcomes resonate with a remarkable blend of accuracy and confidence.

### Data collection instrument and data collection

The CHWs surveyed each participant using a questionnaire/checklist [Table 2] and by observing the practices in their day-to-day life. All data were entered into the Kobo toolbox against the questionnaire by the CHWs. Development of the COVID-19 Knowledge, Attitudes, and Practices (KAP) questionnaire was a thorough and collaborative process designed to ensure its cultural relevance and contextual appropriateness. It involved several key steps, including a review of WHO COVID-19 guidelines, adaptation of pre-existing KAP questions used in other humanitarian settings, incorporation of local knowledge through engagement with community leaders and religious leaders, and pilot testing within a small refugee sample to refine clarity and cultural sensitivity. The final questionnaire was then validated by subject-matter experts and public health professionals familiar with the Rohingya context, while ethical considerations were integrated. This meticulous approach aimed to yield a tool that accurately captures the community’s knowledge, attitudes, and practices related to COVID-19, thus enabling targeted and effective public health interventions in the Rohingya refugee context. The questionnaire/checklist was translated into the Bengali language. The questionnaire comprised 14 questions to assess the respondents’ knowledge. It covers their awareness of COVID-19, ability to identify symptoms, understanding of danger signs like respiratory problems, grasp of transmission modes, recognition of varying symptom severity, knowledge of seeking medical care and testing, awareness of isolation and quarantine protocols, familiarity with preventive measures like hand hygiene and mask-wearing, and whether they’ve heard about the COVID-19 vaccine. 11 and 7 questions were employed, respectively, to evaluate the attitudes and practices of respondents regarding COVID-19. The attitude-related questionnaire explores participants’ perspectives on various aspects of COVID-19. It assesses their perceptions of personal and community risk, the effectiveness of preventive actions like handwashing and mask-wearing, views on staying at home and social distancing, willingness to undergo testing and isolation, belief in the significance of vaccinating, and their optimism regarding pandemic control. The practice questionnaire assesses participants’ tangible COVID-19 actions, spanning both preventive measures (such as mask usage and hand hygiene) and social isolation practices (like avoiding crowds and keeping family members at home). It covers behaviours like frequent handwashing, wearing masks in public, maintaining distance, refraining from touching the face, using proper cough/sneeze etiquette, and inquiring about vaccination for individuals over 55 years. This questionnaire sheds light on participants’ practical responses to the pandemic’s challenges.

### Data analysis

To assess the level of knowledge, attitude and practice of COVID-19 preventive measures, socio-demographic and exposure factors were measured for each variable by using a similar scale developed by Zhong et al. [31]. Using a two-point Likert scale (0 = No, 1 = Yes), CHWs monitored respondents’ actions during the survey period. All responders were asked to respond “Yes” or “No”. Scores were determined by awarding one point for each appropriate answer, with higher scores signifying a greater proficiency level. The Knowledge variable comprises 14 questions, each answered with 1 for "Yes/Correct" or 0 for "No/Don’t know". The scoring for Knowledge ranges from 0 to 14. Scores of 0–4 (0–33%) indicate Poor knowledge, 5–8 (34–67%) denote Moderate knowledge, and 9–14 (68–100%) signify Good knowledge [Table 1]. The Attitude variable consists of 11 questions, using the same response scale. Attitude scores range from 0 to 11, with 0–3 (0–33%) indicating Poor attitudes, 4–7 (34–67%) representing Moderate attitudes, and 8–11 (68–100%) indicating Good attitudes [Table 1]. The Practice variable involves 7 questions, graded as 1 for "Good practice" or 0 for "Poor practice". Practice scores range from 0 to 7. Scoring categories for Practice include Poor (0–3, 0–33%), Moderate (4–5, 34–67%), and Good (6–7, 68–100%) [Table 1]. This scoring framework aids in categorizing participants’ levels of knowledge, attitude, and practice, offering a comprehensive assessment of their engagement with COVID-19-related information and behaviours. The evaluation of internal consistency within a scale encompassing knowledge, attitudes, and practices (KAP), was accomplished through the utilization of Cronbach’s alpha coefficient. The computed alpha value of 0.8680 reveals a commendable level of internal consistency among the KAP items including the average interitem covariance of 3.056569, indicating the extent of correlation between individual items and the overall scale. The bivariate relationship between the socio-demographic and outcome variables was assessed using the Pearson chi-square test and Fisher exact test. Multivariable logistic regression was used to evaluate the status and effectiveness of risk communication and community engagement approaches, inter-activeness, acceptability, and comprehensibility. The logit coefficient is calculated, as well as the 95% confidence interval. The level of statistical significance was set at 5%. All analyses are carried out using the STATA (v-16.0).

**Table 1:** Method of calculating KAP (Knowledge, attitude and practice) score.

Variables	Number of Questions	Score to Answers	Level of Variables
Knowledge	14	1 = Yes/Correct 0 = No/Don’t know	Poor: 0–4 (0–33)
			Moderate: 5–8 (34–67)
			Good: 9–14 (68–100)
Attitude	11	1 = Yes/Correct 0 = No/Don’t know	Poor: 0–3 (0–33)
			Moderate: 4–7 (34–67)
			Good: 8–11 (68–100)
Practice	7	1 = Good practice 0 = Poor practice	Poor: 0–3 (0–33)
			Moderate: 4–5 (34–67)
			Good: 6–7 (68–100)

### Inclusivity in global research

Additional information regarding the ethical, cultural, and scientific considerations specific to inclusivity in global research is included in the Supporting Information (S1 File Inclusivity in global research questionnaire)

### Ethical consideration

The Institutional Review Board (IRB)/Ethical Review Committee (ERC) of North South University, Bangladesh, authorized the protocol for this study (2021/OR-NSU/IRB/0401). All respondents participated voluntarily. Before performing the formal interview, each participant provided written informed consent (mainly thumb imprints), and consent documents were stored separately until the conclusion of the study in a closed filing cabinet. The study adhered to the “no-harm” principle, and there was no legal risk associated with the involvement of the participants. Moreover, local rules and regulations were observed during interactions. Each stage of this investigation was conducted in accordance with the Helsinki Declaration (1964) and its most recent amendment (2013).

## Results

### Sample characteristics of the study population

The demographics of the survey respondents in Rohingya refugee camps in Cox’s Bazar are shown in Table 2. A total of 500 people participated in this study, with 239 men (47.80%) and 261 women (52.20%) making up the majority. The participants in the study were on average 43.98 years old. The study participant’s age categories are distinguished by early, prime, mature, and elderly [32]. This age group is classified by comparing the life expectancies of the host and refugee communities, with the average life expectancy for the Rohingya being 67.36 years and the Bangladeshi being 72.87 years [33]. In the study population, 20.40% were between the ages of 18 and 30, 35.60% were between the ages of 31 and 45, 28.80% were between the ages of 46 and 60, and 15.20% were above the age of 61. 89.20% of the people in the survey were married, 1.80% were single, and 9% were divorced or widowed. Families with fewer than five members accounted for 38.20% of respondents, followed by 34% for families with fewer than seven but more than four members, 21.20% for families with fewer than nine but more than six members, and 6.60% for families with more than nine members. 17.60% of respondents had two children, 23.60% had five or more children, 17% had three children, and 12.60% had one child, which was the same as those who did not have a child. One or more family members under the age of ten years were reported by 63% of respondents, while 26% had family members above the age of 60.

**Table 2:** Descriptive statistics of survey respondents (n =  500).

Sociodemographic Characteristic	Total (n = 500)	
	N	%
Gender		
Female	239	47.80
Male	261	52.20
Agegroup (year)		
18–30 year	102	20.40
31–45 year	178	35.60
46–60 year	144	28.80
> = 61 years	76	15.20
Residence		
Camp 13	100	20.00
Camp 15	100	20.00
Camp 20	100	20.00
Camp 21	100	20.00
Camp 24	100	20.00
Marital Status		
Single	9	1.80
Married	446	89.20
Divorced/Separated/Widowed	45	9.00
People at Household		
< = 4 members	191	38.20
5–6 members	170	34.00
7–8 mebers	106	21.20
> = 9 members	33	6.60
Number of Children		
No child	63	12.60
One child	63	12.60
Two children	88	17.60
Three children	85	17.00
Four children	83	16.60
Five or more children	118	23.60
Family Members above 60 years		
Zero	370	74.00
One	108	21.60
Two or more	22	4.40
Family Members below 10 years		
None	185	37.00
One	78	15.60
Two	120	24.00
Three	83	16.60
Four or more	34	6.80

### Level of knowledge, attitude and practice regarding COVID-19 preventive measures

Table 3 shows the response of the participants on their knowledge, attitude and practice regarding COVID-19 preventive measures. The majority of the participants had an understanding of the different preventive measures for COVID-19. 100% of the participants heard about COVID-19 and more than 90% heard about the COVID-19 vaccine. More than 80% of the participants could explain some symptoms of COVID-19. Around three-fourths of the participants understood how COVID-19 transmits, the necessity of consultation at the health facility for respiratory/COVID-19 symptoms, and the requirement of wearing masks and cough etiquette. Two-thirds of the participants knew that they should wear masks around other people and avoid crowded or closed spaces. Knowledge regarding complications and different severity of COVID-19 was relatively low, around half of the participants positively responded. Around 60% of the participants knew the necessity of providing samples for testing, isolation of positive cases at the isolation and treatment center, and quarantine of contacts of COVID-19.

**Table 3:** Participants’ knowledge, attitude and practices on covid-19 preventive measures.

Knowledge	% Yes	% No
Have they heard about COVID-19?	100	0.00
Can they explain some symptoms of COVID-19? (e.g., fever, cough, respiratory distress, headache, myalgia, runny nose etc.)	82.00	18.00
Do they know dangers/complications of COVID-19? (e.g., respiratory difficulty, death, heart/brain complications etc.)	53.20	46.80
Do they know how COVID-19 transmits? (By coughing, sneezing, contacts, <1 m distancing etc.)	75.20	24.80
Do they know COVID-19 can be presented in varied severity? (e.g., asymptomatic, mild, moderate, severe)	48.20	51.80
Do they know patients with respiratory/COVID-19 symptoms should be consulted at a health facility?	74.20	25.80
Do they know that patients with respiratory/COVID-19 symptoms should give sample (through nasal/oral swab) for testing?	58.80	41.20
Do they know that patients with positive results for COVID-19 should be isolated at a SARI Isolation and Treatment Center?	59.80	40.20
Do they know that contacts (e.g., family members) of a COVID-19 patient should be quarantined (preferably in a Quarantine Facility?)	57.40	42.60
Do they know hand hygiene (using soap and water or alcohol-based hand rub) is one of the measures to prevent transmission?	69.20	30.80
Do they know they should wear mask around other people?	78.20	21.80
Do they know they should avoid crowded or closed spaces?	67.60	32.40
Do they know they should cover their mouth and nose with their bent elbow or tissue when they cough or sneeze?	77.00	23.00
Do they hear about the COVID-19 vaccine?	93.00	7.00
Attitude		
Do you think that COVID-19 can be dangerous for you, your family and neighbors?	71.80	28.20
Do you think that frequent handwashing using soap and water can be an effective measure to prevent or reduce the disease transmission?	66.40	33.60
Do you believe that wearing a mask in public places can be an effective measure to prevent or reduce the disease transmission?	66.20	33.80
Do you think that it is necessary to stay at home and avoid crowded places as much as possible to prevent or reduce the disease transmission?	56.00	44.00
Do you think that it is useful to maintain three feet distance from others not to catch or transmit the disease?	66.00	34.00
Do you agree that you should give sample (via nasal or oral swab), if you are having some symptoms of COVID-19?	58.20	41.80
Do you agree to go to an isolation and treatment center, if you have a positive test result?	58.40	41.60
Do you believe that if some gets positive result for COVID-19, their family should be quarantined to prevent the further transmission?	57.60	42.40
Do you believe the COVID-19 vaccine is essential for us?	85.00	15.00
Are you willing to be vaccinated if the vaccine is offered to you?	89.80	10.20
Do you agree that COVID-19 will finally be successfully controlled?	80.00	20.00
Practice		
Frequently wash their hands with soap and water	30.80	69.20
Wearing a mask in the public place	30.20	69.80
Family is staying at home, if not very critical to go outside	30.00	70.00
Trying to maintain distance with others	37.40	62.60
Not touching mouth, nose, or eye with uncleaned hands	32.20	67.80
Covering mouth by elbow or handkerchief while coughing sneezing	40.80	59.20
Have you been vaccinated? (Applicable if age is >55 Years)	69.60	30.40

More than 70% of the participants perceived COVID-19 as dangerous for them and their families. Two-thirds of the participants had positive attitudes toward handwashing and wearing masks in public places and maintaining social distancing. The majority of the participants had positive attitudes toward the essentiality of the COVID-19 vaccine (85%) and administering the COVID-19 vaccine (89.8%). Below 60% of the participants agreed to avoid crowded places, give samples if having symptoms, admission into an isolation and treatment center and quarantine the family members in case of a positive result. 80% of the participants agreed that COVID-19 could finally be controlled.

In comparison to knowledge and attitude, the practice of COVID-19 different preventive measures among the participants was found low. Below one-third of the participants frequently washed their hands, wore masks in public places, stayed home if not critical, and avoided touching their mouth, nose, or eye with uncleaned hands. Only 37% of participants tried to maintain distance from others and 40% practiced cough etiquette. However, around 70% of the participants, who were over 55 years, received a dose of COVID-19 vaccine.

Table 4 shows the results of the study in terms of level of knowledge, attitude, and practice. The findings suggest that many respondents (70.80%) had a good understanding of COVID-19, with only a minority (16.0%) having a poor knowledge level. Only 22.20% of respondents had a good level and 37.02% had an average level of attitude toward maintaining COVID-19 preventive measures. One-fifth of the participants (22%) had a poor level of attitude. In terms of practice level, it was also found that only 7.00% of the respondents had a good level of preventive practices, while the majority of respondents (61.80%) had a poor level of practice. In summary, the majority of the community had a good level of knowledge or awareness of COVID-19 and an average to a good level of attitude, however, a significantly low level of practice toward the preventive measures.

**Table 4:** Knowledge, attitude and practice regarding COVID-19 preventive measures.

Variable	Overall Mean (+/- SD)	Poor	Average	Good
Knowledge	9.93 (2.95%)	80 (16.00%)	113 (22.60%)	354 (70.80%)
Attitude	7.55 (2.51%)	111 (22.20%)	186 (37.20%)	111 (22.20%)
Practice	2.71 (1.58%)	309 (61.80%)	201 (40.20%)	35 (7.00%)

Table 5 illustrates the association between the socio-demographic distribution of the respondents and knowledge, attitude, and practice regarding COVID-19 preventive measures. In terms of age, except for elderly people, almost all age groups had nearly the same percentage of responders (60~70%) with good awareness of COVID-19 infection, prevention, and control strategies. Good attitude toward COVID-19 preventive measures was found similar among all age groups, between 38–44%. While the prevalence of the good practice of COVID-19 preventive measures was found low in all age groups (2 ~ 11%), it is lower (3.92%) among the early working age group (18–30 years) and lowest (2.63%) among elderly (>/ = 61 years). It was found that almost an identical proportion (~62%) of the male and female participants have a good level of knowledge. In terms of the gender distribution of good level of practice and attitude, a bit lower prevalence was found among female participants in comparison to males.; 38.08% of the female participants had a good attitude which is lower in comparison to the male (42.15%), and 6.28% of the female participants had a good practice level which is lower in comparison to the male (7.66%). We found that those who were separated/widowed (53.33%) had less understanding of COVID-19 than the single (77.78%) or married respondents (62.33%). Single respondents (66.67%) had a more positive attitude than married (39.91%) or separated/widowed respondents (37.78%). Positive attitudes toward preventive measures were found lower among married (39.91%) individuals and divorced/separated/widowed in comparison to singles (66.67%). Good practice level was also found lower among married (7.40%) and lowest among divorced/separated/widowed (2.22%) in comparison to singles (11.11%). We observed that 56.02% of respondents from small/nuclear families had a good level of knowledge, while 84.50% of respondents from very big families had a good level of knowledge. Good attitude level remains within 38–45% among respondents from different family sizes. However, good practice level was noted among 9.42% of respondents from small families and 3–4% among large and very large families. Through bivariate analysis, the study found a significant association between family size and knowledge level (Fisher’s exact 0.02), however, no statistical association of knowledge, attitude and practice were found with gender and marital status.

**Table 5:** Distribution of respondents and ‘knowledge, attitude and practice’ scores of COVID-19 across demographics in fdmn camps (n = 500).

Variable	Determinants	Knowledge level			Chi-square	p-value
		Poor	Average	Good		
Agegroup	18–30 year	13 (12.75%)	20 (19.61%)	69 (67.65%)	9.37	0.104
	31–45 year	26 (14.61%)	36 (20.22%)	116 (65.17%)		
	46–60 year	23 (15.97%)	33 (22.92%)	88 (61.11%)		
	> = 61 years	18 (23.68%)	22 (28.95%)	36 (47.37%)		
Gender	Female	39 (16.32%)	53 (22.18%)	147 (61.51%)	0.03	0.902
	Male	41 (15.71%)	58 (22.22%)	162 (62.07%)		
Marital Status	Single	1 (11.11%)	1 (11.11%)	7 (77.78%)	0.094 (Fisher’s exact)	
	Married	75 (16.82%)	93 (20.85%)	278 (62.33%)		
	Divorced/Separated/Widowed	4 (8.89%)	17 (37.78%)	24 (53.33%)		
Family Size	< = 4 members	30 (15.71%)	54 (28.27%)	107 (56.02%)	0.026 (Fisher’s exact)	
	5–6 members	32 (18.82%)	31 (18.24%)	107 (62.94%)		
	7–8 members	17 (16.04%)	22 (20.75%)	67 (63.21%)		
	> = 9 members	1 (3.03%)	4 (12.12%)	28 (84.85%)		
		Attitude level				
		Poor	Average	Good		
Age	18–30 year	21 (20.59%)	36 (35.29%)	45 (44.12%)	8.15	0.207
	31–45 year	31 (17.42%)	76 (42.70%)	71 (39.89%)		
	46–60 year	38 (26.39%)	50 (34.72%)	56 (38.89%)		
	> = 61 years	23 (30.26%)	24 (31.58%)	29 (38.16%)		
Gender	Female	49 (20.50%)	99 (41.42%)	91 (38.08%)	3.60	0.105
	Male	64 (24.52%)	87 (33.33%)	110 (42.15%)		
Marital Status	Never married	0.00 (0%)	3 (33.13%)	6 (66.67%)	0.204	
	Married	106 (23.77%)	162 (36.32)	178 (39.91%)		
	Divorced/Separated/Widowed	7 (15.56%)	21 (46.67%)	17 (37.78%)		
Family Size	Small (</ = 4)	48 (25.13%)	69 (36.13%)	74 (38.74%)	2.48	0.801
	Medium (5–6)	36 (21.18%)	62 (36.47%)	72 (42.35%)		
	Large (7–8)	24 (22.64%)	42 (39.62%)	40 (37.74%)		
	Very Large (>/ = 9)	5 (15.15%)	13 (37.20%)	15 (45.45%)		
		Practice level				
		Poor	Average	Good		
Agegroup	18–30 year	81 (79.41%)	17 (16.67%)	4 (3.92%)	0.092 (Fisher’s exact)	
	31–45 year	121 (67.98%)	44 (24.72%)	13 (7.30%)		
	46–60 year	94 (65.28%)	34 (23.61)	16 (11.11%)		
	> = 61 years	58 (76.32%)	16 (21.05%)	2 (2.63%)		
Gender	Female	166 (69.46%)	58 (24.27%)	15 (6.28%)	1.34	0.511
	Male	188 (72.03%)	53 (20.31%)	20 (7.66%)		
Marital Status	Single	6 (66.67%)	2 (22.22%)	1 (11.11%)	0.568 (Fisher’s exact)	
	Married	316 (70.85%)	97 (21.75%)	33 (7.40%)		
	Divorced/Separated/Widowed	32(71.11%)	12 (26.67%)	1 (2.22%)		
Family Size	< = 4 members	124 (64.92%)	49 (25.65%)	18 (9.42%)	0.310 (Fisher’s exact)	
	5–6 members	125 (73.53%)	33 (19.41%)	12 (7.06%)		
	7–8 members	81 (76.42%)	21 (19.81%)	4 (3.77%)		
	> = 9 members	24 (72.73%)	8 (24.24%)	1 (3.03%)		

The scores of KAP were separated into three classes and placed as the dependent variable using the quartile as a cutoff point. The model is comprised of three continuous processes that aid in the explanation of human behaviour residing in the camps. Table 6 illustrates in the multivariable logistic regression analyses, there is no statistical relevance with gender and marital status on KAP scale in terms of knowledge, attitude and practice regarding COVID-19 preventive measures. Association was found between knowledge and practice level and age group–elderly age group (>/ = 61 years) had less level of knowledge (AOR 0.42, P value 0.058) and the late mid-age group (46–60 years) had better practice level (AOR 2.67, P value 0.001). A significant association was also found between good knowledge level and medium family size (5–6 members) (P value, 0.028).

**Table 6:** Significant factors associated with knowledge, attitude and practice regarding COVID-19 preventive measures.

Variable	Traits	aor*	knowledge, 95% ci		p>|z|	aor*	attitude, 95% ci		p>|z|	aor*	practice, 95% ci		p>|z|
			lower	upper			lower	upper			lower	upper	
Gender	female	Reference				-	-	-	-	-	-	-	-
	Male	1.10	0.65	1.86	0.712	0.81	0.51	1.30	0.396	0.84	0.55	1.28	0.436
Age group	18–30 year	Reference				-	-	-	-	-	-	-	-
	31–45 year	0.80	0.36	1.77	0.591	1.19	0.60	2.34	0.615	**2.56**	**1.35**	**4.86**	<0.001
	46–60 year	0.79	0.35	1.73	0.560	0.81	0.42	1.55	0.535	**2.67**	**1.40**	**5.08**	<0.001
	>/ = 61 years	0.42	0.17	1.02	0.058	0.63	0.29	1.37	0.249	1.51	0.68	3.36	0.302
Marital status	Never married	Reference				-	-	-	-	-	-	-	-
	Married	0.28	0.02	2.74	0.276	0.49	0.19	1.23	0.132	0.34	0.07	1.63	0.181
	Divorced/separated/widowed	0.80	0.06	10.29	0.865	-	-	-	-	0.26	0.04	1.52	0.137
Family size	< = 4	**Reference**				**-**	**-**	**-**	**-**	**-**	**-**	**-**	**-**
	5–6	0.38	0.16	0.90	0.028	0.49	0.22	1.06	0.072	0.65	0.31	1.35	0.250
	7–8	0.71	0.24	2.06	0.533	0.78	0.30	2.01	0.619	0.51	0.20	1.27	0.150
	>/ = 9	5.28	0.56	49.90	0.146	1.60	0.42	6.14	0.487	0.67	0.20	2.22	0.523

* Adjusted odds ratio

## Discussions

This study aimed to assess the level of knowledge, attitude, and practice concerning COVID-19, as well as the perceptions and importance of sociodemographic characteristics that influence KAP. Overall, the study found that most of the participants had a good level of knowledge on COVID-19 and its preventive measures, an average to a good level of attitude and a significantly low level of practice toward the preventive measures. This can be comparable to the KAP among Afgan refugees in Pakistan who were found having a good level of knowledge (78.55%) and positive attitude (80.1%) and lower level of practice (58.5%) of the preventive measures [14]. Research among the Syrian refugees found having moderate level of knowledge (mean 3.37) and practice (3.25) [15], which is dissimilar to the findings in our study having good level of knowledge but a poor level of practice.

According to the author’s knowledge, this is the second study that evaluates the KAP of Rohingya refugees in relation to COVID-19, allowing us to assess and compare our findings with those of a previous study conducted in Rohingya refugee camps and other refugee-related KAP studies. The results of this study indicate that the mean scores for knowledge and attitude were 9.93 (out of 14) and 7.55 respectively (out of 11). This reflects an increase in knowledge and attitude among Rohingya refugees compared to the results of previous research done at the beginning of the pandemic [15], which indicated the mean score of 5.8 (out of 10) for knowledge and 2.2 (out of 5) for attitude. When it comes to quantifying the level of practice, our study found a mean score of 2.71 (out of 7) indicating that practice is not improved as much as knowledge and attitude. The practice level, however, is better than that found in the prior study; the mean practice score was 0.9. (Out of 5) [29]. The improvement of knowledge, attitude, and practice can be linked to the extensive risk communication and community engagement (RCCE) activities undertaken by humanitarian agencies [34, 35]. The RCCE strategies of IOM and the health sector for COVID-19 include dissemination of information and engagement of community through household visits (with proper distancing), distribution of promotional materials, small group and courtyard meetings, and engagement of community leaders [36, 37].

Although the participants had overall good knowledge on the majority of the areas in COVID-19 preventive measures, it was found that only around half of the respondents had awareness of the complications and severity of COVID-19. Inadequate awareness on the severity and complications of COVID-19 might result in inadequate practice of the preventive measures. Therefore, further review of existing RCCE materials is needed to adequately address the severity and complications of COVID-19.

Although we found overall good knowledge and a positive attitude towards the preventive measures, especially for avoiding crowds, wearing masks in public, practicing physical distancing, and maintaining hand hygiene, relatively few people were found actually putting these attitudes into practice. According to the Refugee Influx Emergency Vulnerability Assessment (REVA-5) undertaken by the UN agencies, 95% of the Rohingya families are entirely dependent on humanitarian assistance [38]. Practicing COVID-19 preventive measures require resources, for example, soap, water, masks, etc. As reported by Joint Multisector Needs Assessment (J-MSNA) [39], one-third of the households in the Rohingya refugee camps reported not having enough water and roughly half of the households reported to have faced problems related to latrines. Therefore, limited access to resources required for COVID-19 practices could be related to the low level of practice. The Rohingya refugees are living in overcrowded shelters and need to use common latrines and bathing cubicles, therefore, some of the preventive measures, specially, social distancing and staying at home, may not be feasible in their setting. A high rate of illiteracy, 75% among adult Rohingya women and 61% among adult Rohingya men [40] could also be a contributory factor for the low level of practice.

Our study found a significant association between knowledge level and age group–the older age group (>61 years) was found to have less level of knowledge in comparison to the age group (AOR 0.42, P value 0.058). This contradicts the findings of the first KAP study, which found no significant difference in KAP score based on age. However, another study in the same setting found that there was little fear of COVID-19 among the older Rohingya refugees, which could be related to their lack of awareness of the severity of the disease [41]. Older persons are disproportionately affected by emergencies and may have limited access to information. In 2021, Lebrasseur et al. found that the COVID-19 pandemic had a major impact on vulnerable populations, notably older people, who often experience loneliness, age discrimination, and anxiety [42]. Therefore, the low level of knowledge on COVID-19 preventive measures among the Rohingya older people could be related to their lack of access to information resulting from self-isolation/quarantine, limited social network, lack of connection with family members and inadequate social and psychosocial support [43]. While the existing risk communication and community engagement interventions might have contributed in extensive awareness raising among the general population, the strategies may not adequately addressing the vulnerability of the older persons in terms of accessing information. Further research in this area could reveal the specific challenges of older persons in accessing the information, thereby, informing the RCCE stakeholders for adapting necessary strategy.

Although there was a general low level of practice of COVID-19 measures, we found that a significant number of participants over the age of 55, roughly two-thirds, received the COVID-19 vaccine. This study accounted vaccination for people aged over 55 because during the data collection vaccination was only available for this age group. This can be linked to the fact that to increase vaccine acceptance and uptake, Community Health Working Group (CHWG) implemented extensive risk communication and community engagement strategies along with evidence-based initiatives [44, 45].

### Limitations and future directions

The design of this study was cross-sectional; hence, causal inferences may not always be made. The study found that many of the participants had a good level of knowledge and an average level of attitude, however, a significantly low level of practice of the COVID-19 preventive measures. While the study provides a numerical analysis, further qualitative investigations is required to understand the drivers and barriers of this situation. The quantitative findings of the study on different areas and levels of knowledge, attitude and practice together with proposed qualitative investigation should be taken into consideration for updating the risk communication and community engagement strategy for COVID-19 and future similar outbreaks.

## Conclusions

The study found that there is an improved level of knowledge and attitude toward COVID-19 preventive measures among the Rohingya refugees in Cox’s Bazar. This could be contributed to the extensive risk communication and community engagement strategies by the humanitarian partners. However, it was found that the practice level, although improving in comparison to the baseline, is significantly poor. A significant association was found between age group and knowledge level—elderly age group had less level of knowledge regarding COVID-19 preventive measures. Further initiatives should be undertaken to identify the causes behind the poor practice level, especially how the policies and strategies as well as the local context, resources and social factors are related to the practice. We urge that the health and RCCE partners consider the findings of this study to develop future risk communication and communication engagement strategies for COVID-19 and future outbreaks. While robust initiative should be taken to improve the practice level, attention should also be given to improving some areas of knowledge and attitude especially, the complications and severity of COVID-19. Factors behind poor level of knowledge among older persons should be explored and addressed.

## Supporting information

S1 Dataset This contains the full dataset of the study. (XLSX)

S1 File Inclusivity in global research questionnaire. Additional information regarding the ethical, cultural, and scientific considerations specific to inclusivity in global research.
(DOCX)

## Abbreviations

AWD: Acute Watery Diarrhoea
CHW: Community Health Worker
CI: Confidence Interval
CwC: Communication with Community
ERC: Ethical Review Committee
FDMN: Forcefully Displaced Myanmar Nationals
IOM: International Organization for Migration
IRB: Institutional Review Board
ISCG: Inter Sector Coordination Group (ISCG)
KAP: Knowledge, Attitude, and Practice
MERS-CoV: Middle East Respiratory Syndrome Coronavirus
NSU: North South University
RCCE: Risk Communication and Community Engagement
SARS-CoV-2: Severe Acute Respiratory Syndrome Coronavirus 2
WASH: Water, Sanitation and Hygiene
WG: Working Group
WHO: World Health Organization
